# Bibliometric analysis: Research trends of acupuncture treatment to cognitive impairment in recent 15 years

**DOI:** 10.3389/fpsyg.2022.935053

**Published:** 2022-10-13

**Authors:** Chen-Chen Nie, Kai-Qi Su, Jing Gao, Xiao-Lei Song, Zhuan Lv, Jie Yuan, Meng Luo, Xiao-Di Ruan, Yong-Fu Fan, Ming-Yue Yu, Shi-Kui Qi, Xiao-Dong Feng

**Affiliations:** ^1^Department of Rehabilitation Medicine, Henan University of Chinese Medicine, Zhengzhou, China; ^2^Rehabilitation Center, The First Affiliated Hospital of Henan University of Chinese Medicine, Zhengzhou, China

**Keywords:** acupuncture, cognitive impairment, bibliometric analysis, CiteSpace, VOSviewer

## Abstract

**Objectives:**

Acupuncture therapy has been used for cognitive impairment-related diseases, however, there are still few studies on the overall trend of acupuncture therapy on cognitive impairment based on bibliometric analysis. The purpose of this study was to explore the research trend of the impact of acupuncture on cognitive impairment in the past 15 years, analyze the research trends and hotspots, and provide new ideas and theoretical basis for future research directions.

**Methods:**

From the Web of Science Core Collection (WoSCC), the relevant literature on the treatment of cognitive impairment with acupuncture from 2007 to 2022 was retrieved. Then, based on the CiteSpace and VOSviewer software of the Java platform, the cooperation between countries and institutions in this field, the co-citation of journals and documents, and the cooperation between authors and authors, etc. were analyzed. In addition, the co-occurrence and burst analysis of keywords are also carried out, and a visual knowledge map is drawn.

**Results:**

As of August 08, 2022, a total of 394 records related to the treatment of cognitive impairment with acupuncture were identified. The analysis results show: The number and rate of annual publications have steadily increased, with some fluctuations from year to year. The countries that contribute the most to this field are China and the USA. Among them, Beijing University of Chinese Medicine and Capital Medical University are tied for first place in terms of the number of published papers. Tao Jing is the most prolific author and the number one cited author.

**Conclusions:**

The number of publications on acupuncture for cognitive impairment is expected to increase rapidly in future research, suggesting a bright future for the field. Future research hotspots will focus on pain, injury, protocol, diagnosis, guidelines, etc. It is also necessary to strengthen cross-regional and cross-country cooperation among various academic groups.

## Introduction

Cognitive impairment refers to the abnormal processing of higher brain functions related to learning and memory and thinking and judgment, resulting in serious impairment of attention, learning and memory, executive function, language function and social cognitive function (Owens et al., 2020), it can affect the physical health and social function of patients to varying degrees, reduce their quality of daily life, and in severe cases can be life-threatening (Powers et al., 2013). Cognitive impairment can be caused by neurodegenerative diseases, cardiovascular diseases, cerebrovascular diseases, nutritional and metabolic disorders, trauma, infections, and drugs. With the acceleration of the global aging process, changes in the population structure have gradually increased the proportion of the population with cognitive impairment, currently, it has become one of the hotspots in modern medicine and geriatrics research, and the number of related studies is showing a trend of continuous growth. Relevant research has shown that people's acceptance of complementary and alternative medicine (CAM) is gradually increasing worldwide, such as Chinese herbal medicine (Zeng et al., 2015), acupressure (McFadden et al., 2011) and traditional Chinese exercises, etc (Wu et al., 2021). Acupuncture as a component of CAM is safe and effective with few side effects, which has played a definite role in the treatment of cognitive impairment (Ghafoor et al., 2019; Su et al., 2019; He et al., 2021).

Bibliometrics is a research method based on mathematics and statistics, focusing on a systematic analysis of the characteristics of literature, and used to summarize the development status of a certain research field, show future trends, and provide references for basic and clinical research. In addition to describing and predicting future trends in a specific field, it is also possible to compare contributions from various authors, institutions, countries, and journals. This analytical approach plays a vital role in developing guidelines, understanding research hotspots, and assessing research trends (Guler et al., 2016). Currently, bibliometric analysis is used to investigate overall trends in acupuncture research (Ma et al., 2016; Li et al., 2019), Researchers have used this method to begin studying diseases of the digestive system (Huang et al., 2019), cancer (Zhang et al., 2020), rheumatic diseases (Liang et al., 2020), and diseases of the cardiovascular system (Martynov et al., 2020). However, studies on the general trends of acupuncture in the treatment of cognitive impairment based on bibliometric analysis are still few.

This article discusses the research hotspots and development trends in the field of acupuncture treatment of cognitive impairment in the past 15 years, and the scientific knowledge map was drawn using CiteSpace and VOSviewer software. Its purpose is to provide a basis for scientific research in neurological disorders.

## Methods

### Data source and search strategy

#### Data source

The Web of Science Core Collection (WOSCC) database was used as the data source for retrieval, and the retrieval date was 2022-08-08.

#### Search strategy

Using the advanced search capabilities of the Web of Science database, formulated search formula: TS= ((acupuncture^*^ OR acupuncture Therapy^*^ OR acupuncture points^*^ OR body acupuncture^*^ OR auricular acupuncture^*^ OR electroacupuncture^*^ OR moxibustion therapy^*^)AND (cognitive dysfunction^*^ OR mild cognitive impairment ^*^ OR mci^*^ OR cognitive impairment^*^ OR cognitive decline^*^ OR mental deterioration^*^)). The index has been limited to SCI-EXPANDED, SSCI; the document type are limited to papers and review papers; the publication time is limited to 2007-08-08–2022-08-08.

Conference papers, abstract compilations, training notices, news reports, duplicate publications and other documents have been excluded.

### Data processing

**Figure 1:**
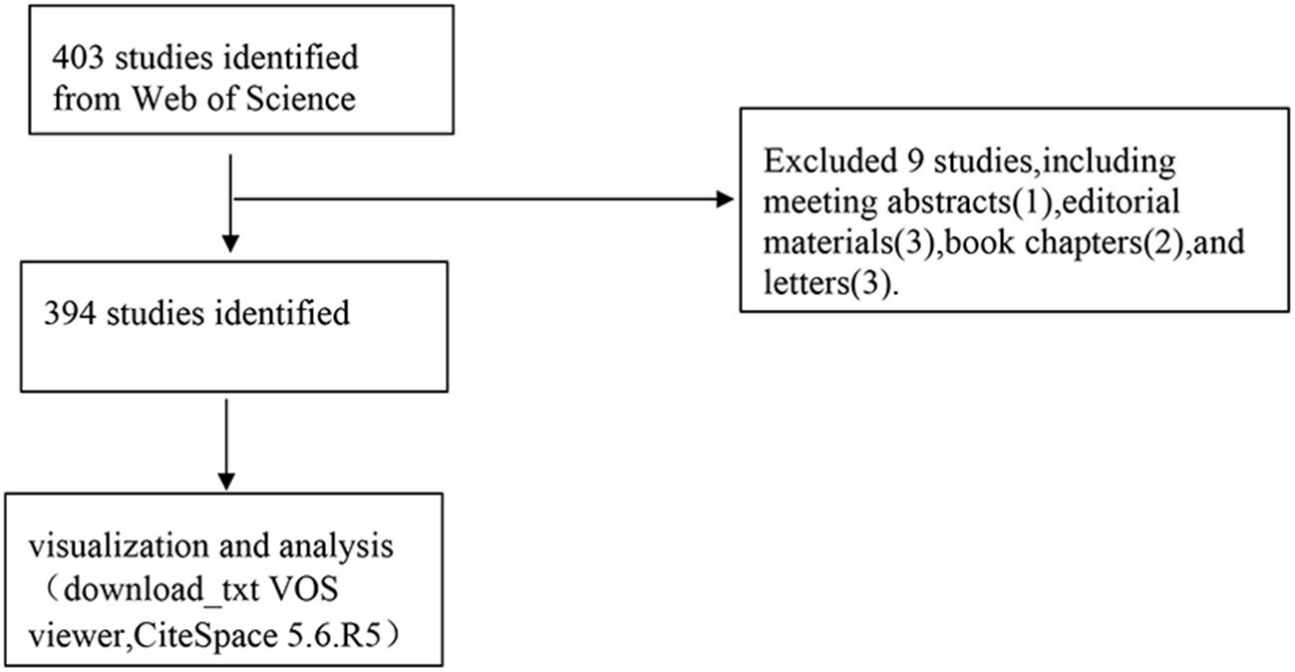
Flowchart of literature screening.

All valid papers retrieved from the Web of Science Core Collection were saved as plain text files and saved in download_txt format in Figure 1. Data were independently extracted from selected publications by the first author and, in the case of disputes, mediated by the second author (Kai-Qi Su).

### Bibliometric analysis

Visual analysis was performed on all files filtered out and saved in download_txt format using VOSviewer and CiteSpace software.

CiteSpace (Version 5.6.R5, Drexel University, Chao-mei Chen) is a visualization tool developed by Dr. Chen Chao-mei that runs under the Java system and is used for bibliometrics (Chen, 2004). The analysis results can be displayed from macro to micro levels for each element of literature research, helping scholars to understand the development trends in this field more intuitively and comprehensively. It presents the structure, laws, and distribution of scientific knowledge maps using data mining, information analysis and map drawing. The knowledge map is a new subfield of information technology, it is used to visually visualize research hotspots and evolutionary processes, and predict the development trend of each field, and is widely regarded as an excellent scientometric analysis tool and a good choice for bibliometric analysis (Liu et al., 2014).

VOSviewer (Version 1.6.14, Leiden University, van Eck NJ) is a scientometrics network analysis software developed by the Center for Scientific and Technological Research, Leiden University, The Netherlands. It provides visual analysis and creates maps from network data, not only can network maps of countries and institutions, journals, authors, and keywords be constructed but items in these networks can be connected by co-citation and co-occurrence, etc. The VOSviewer software provides three visualization maps: network, overlay and density visualization (van Eck and Waltman, 2010). The network in this study was constructed based on co-authorship of authors, co-citation analysis of authors, and co-occurrence of keywords. Keywords that appeared more than 10 times were also included in a co-occurrence network analysis to identify important terms in research on acupuncture for cognitive impairment.

In order to gain an in-depth understanding of the international research trends and hotspots about acupuncture treatment of cognitive impairment, improve the accuracy of software algorithms for research results, reduce the variance of analysis results. In this paper, the two bibliometric software CiteSpace and VOSviewer are combined to research and analyze the relevant literature on acupuncture treatment of cognitive impairment in the Web of Science database from 2007 to 2022, generate a visual scientific knowledge map, and analyze the relevant data such as the distribution of national and regional cooperation, journals and cited journals, authors and author cooperation, keywords and emerging words, to discover the research status and trends in this field, it can also provide a reference for basic research and clinical treatment of cognitive impairment.

## Results and analysis

### Published products and time trends

**Figure 2:**
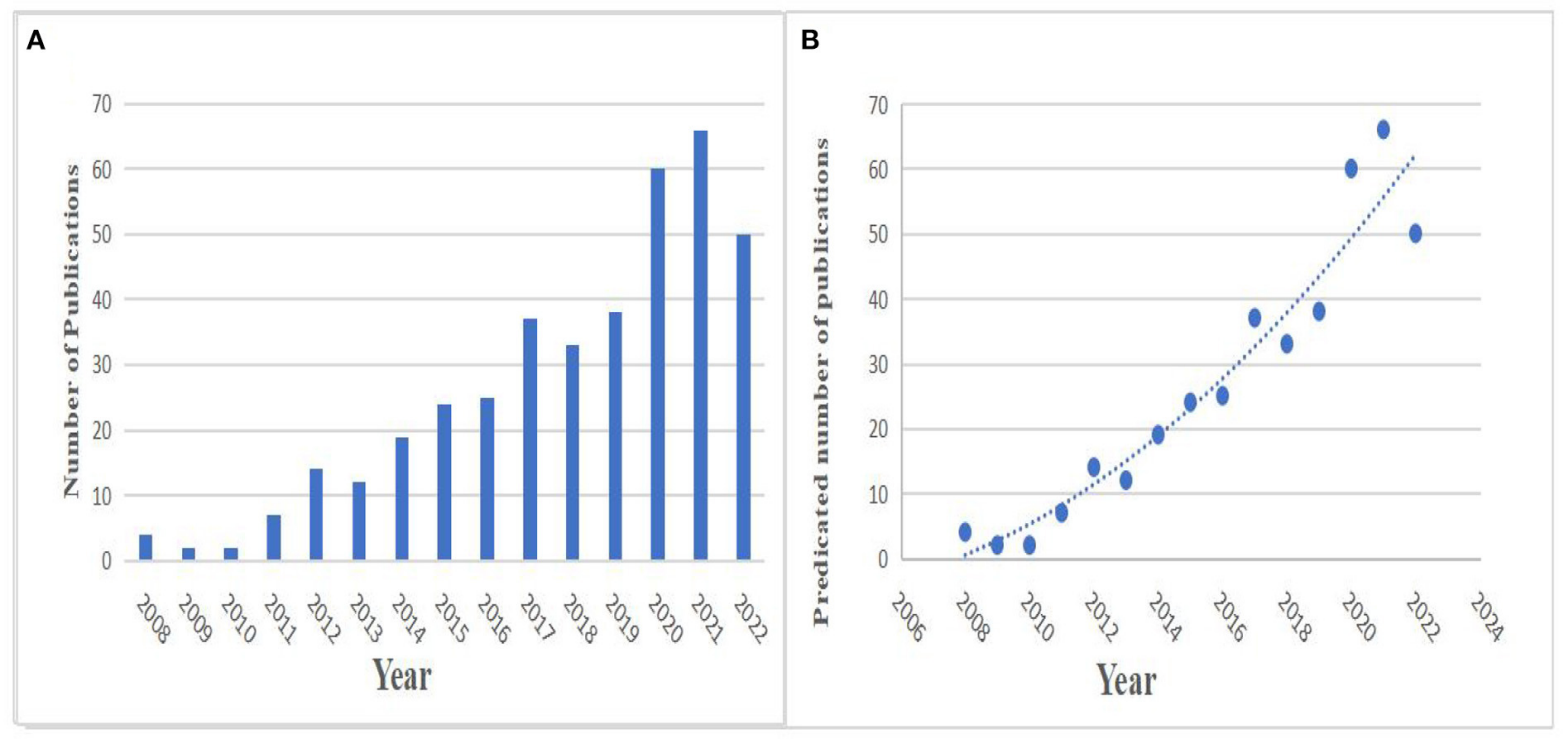
Global trends in publications on acupuncture for cognitive impairment. **(A)** Number of publications over the past 15 years. **(B)** Display publication growth trends and curves, and forecasts for future publications.

From four articles in 2007 to 50 articles in 2022, it can be seen that in the past 15 years, the number of articles related to acupuncture treatment of cognitive impairment has increased steadily, but there is a certain fluctuation between years in Figure 2A. A logistic regression model was used to create a time curve of the number of publications that could predict future trends in Figure 2B. This time curve shows that the field is currently in a phase of steady growth in global publication output. Furthermore, the growth rate is expected to increase rapidly over the next decade.

### Distribution of country and institution

**Figure 3:**
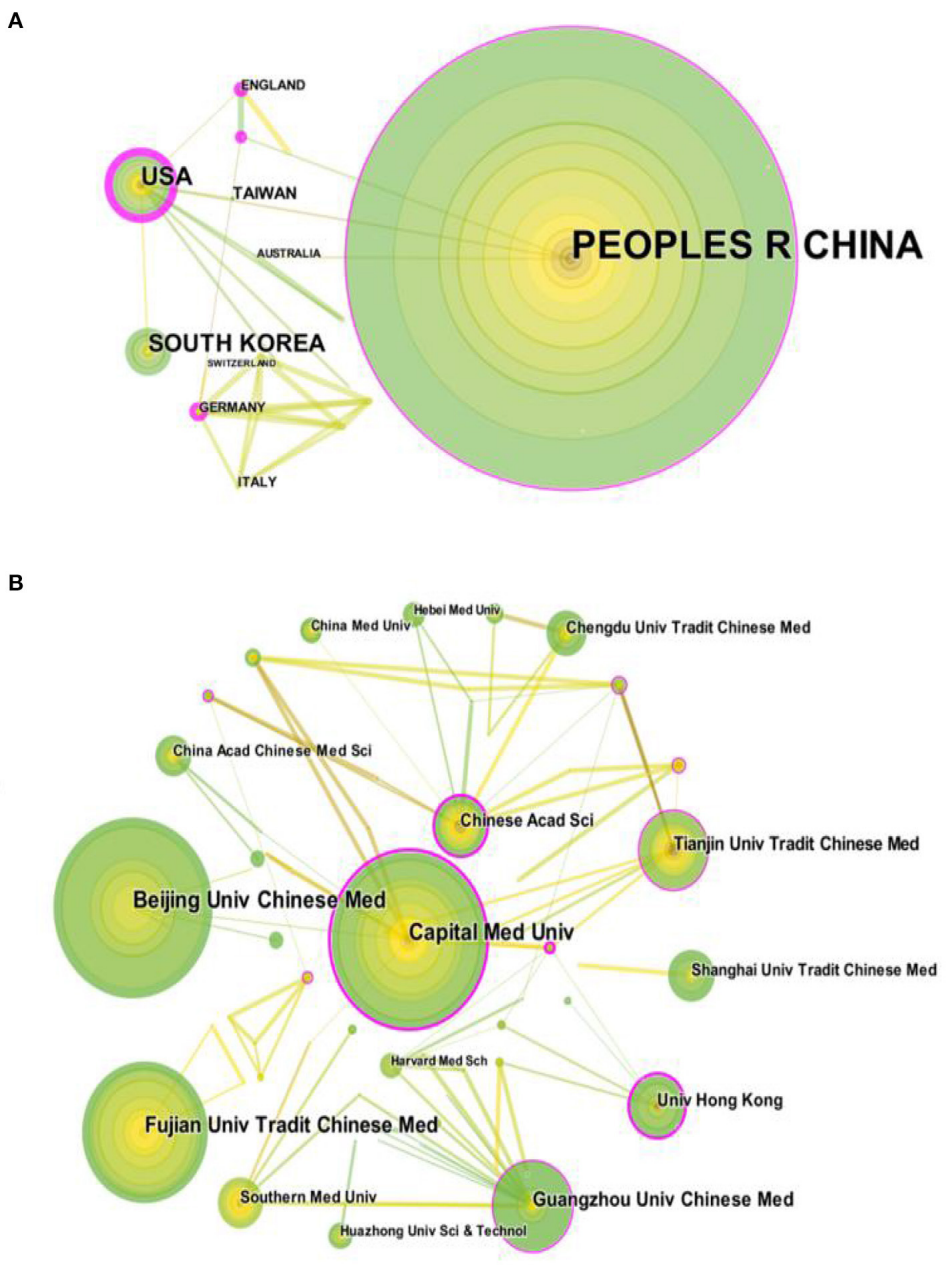
Cooperative network of countries/regions and institutions that publish relevantly literature **(A)** Co-authoring network map among countries/ regions acupuncture treats cognitive impairment. **(B)** Network map co-authored between institutions. In a network map, a dot represents a country/institution, and a line between 2 dots represents a partnership, a wider line indicates a stronger relationship.

CiteSpace analyzed 27 countries and regions that contributed to publications on acupuncture for cognitive impairment in Figure 3A. It can be seen from Table 1 that China (257 papers), the USA (39 papers), South Korea (33 papers), Germany (9 papers) and England (6 papers) were the top five productive countries and regions. In terms of centrality (purple), the top three countries are the USA (0.63), England (0.47), China (0.1), this finding suggests that these countries may have played an important role in research in this area.

**Table 1:** Top five countries/regions with the number of published articles.

**Rank**	**Country**	**Counts (*n*)**	**Centrality**
1	China	257	0.1
2	The USA	39	0.63
3	South Korea	33	0
4	Germany	9	0
5	England	6	0.47

CiteSpace was used to analyze the 275 institutions that contributed to the field in Figure 3B. Beijing University of Chinese Medicine (38 papers), Capital Medical University (38 papers), Fujian University of Traditional Chinese Medicine (31 papers), Guangzhou University of Chinese Medicine (20 papers) and Tianjin University of Traditional Chinese Medicine (18 papers) are the top five producing institutions. In terms of centrality, the top three institutions are the Chinese Academy of Sciences (0.27), Beijing Huairou University of Traditional Chinese Medicine (0.23) and Hong Kong University (0.23).

### Distribution of journals and co-authored academic journals

In the related research on acupuncture treatment of cognitive disorders, 394 articles were published in 134 academic journals. The top five journals that published articles on acupuncture treatment of cognitive impairment are shown in Table 2. The *Evid-Based Complem Alternm* has the largest number of published articles (34 papers, 10.4% of all articles).

**Table 2:** Top five journals and cited journals related to acupuncture treatment of cognitive impairment.

**Rank**	**Cited journals**	**Citations**	**Rank**	**Popular journals**	**Records**
1	*BMC Complem Alternm*	395	1	*Evid-Based Complem Alternm*	34
2	*Evid-Based Complem Alternm*	330	2	*Medicine*	24
3	*Plos One*	285	3	*Acupuncture Med*	18
4	*Acupuncture Med*	232	4	*Trials*	17
5	*Molecular Neurobiology*	184	5	*Neural Regeneration research*	16

**Figure 4:**
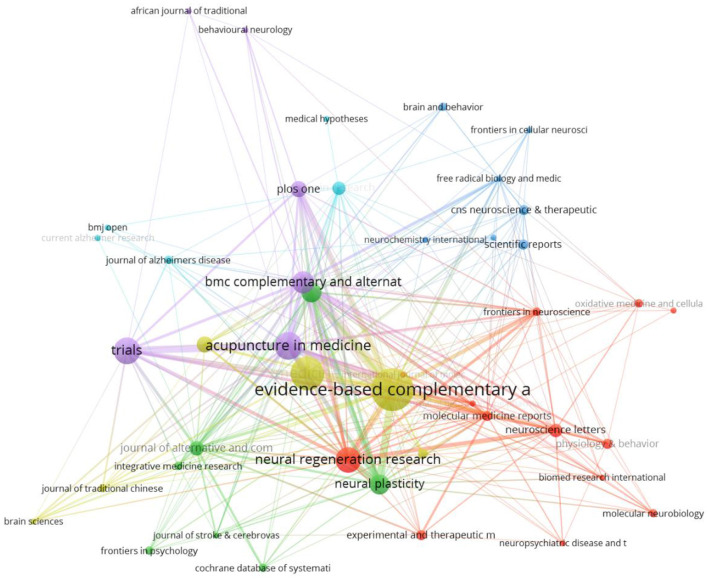
Network map of cited journals.

From the citing journal graph in Figure 4 generated by VOSviewer, we found that *BMC Complem Alternm* ranked first in the frequency of citing journals (395 times), followed by *Evid-Based Complem Alternm* (330 times), *Plos One* (285 times), *Acupuncture Med* (232 times) and *Molecular Neurobiology* (184 times).

**Figure 5:**
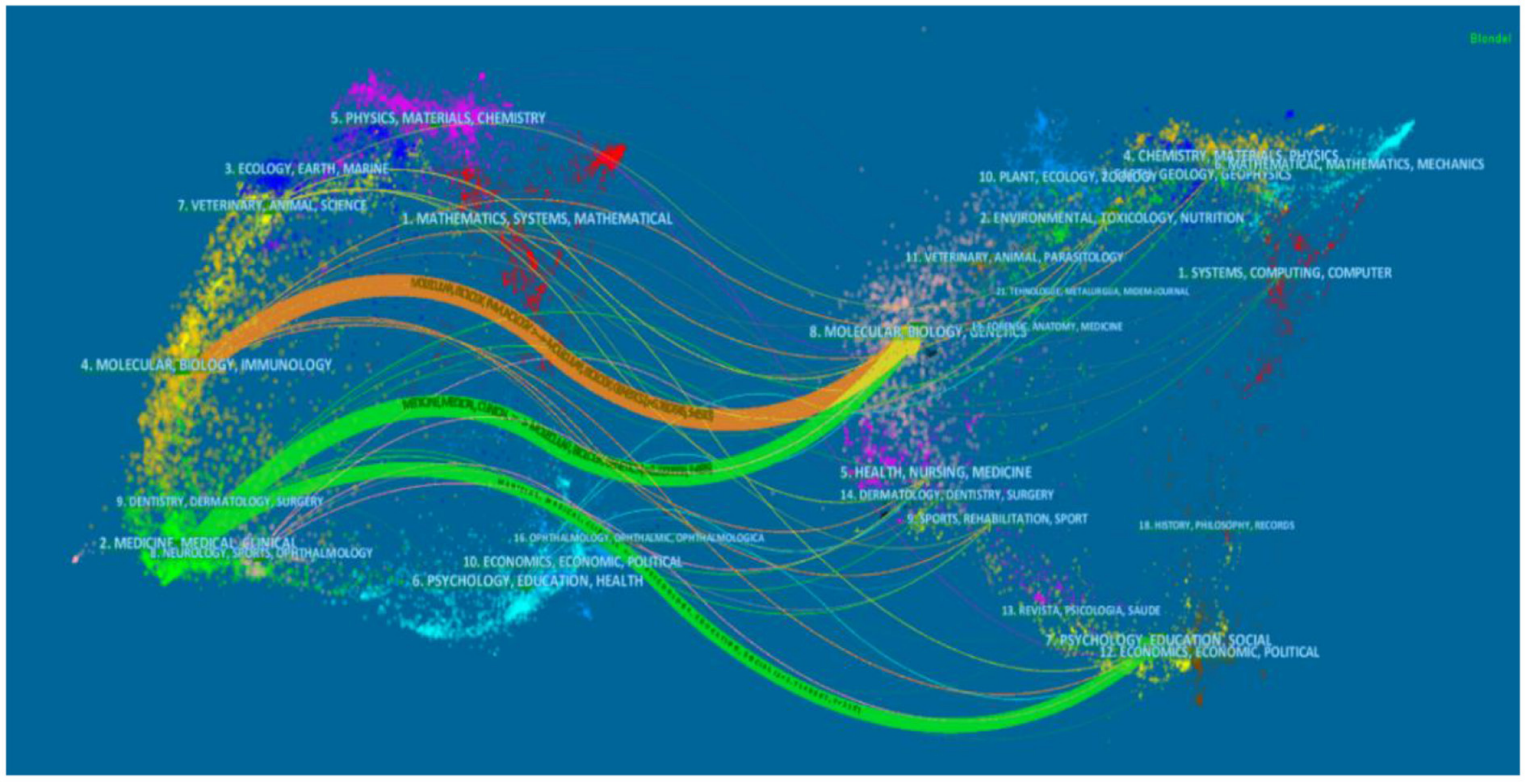
Dual-map overlay of journals.

A journal dual map overlay produced by CiteSpace shows the distribution of relationships between journals. The labels on the map represent the different research topics covered by all journals and can reveal trends in a research topic as a whole. The different colored paths between them indicate the referenced relationship, the width of the connection paths is proportional to the frequency of z-score scale citations. As shown in Figure 5, there are currently three main reference paths on the map, including an orange path and two green paths. Orange paths indicate studies published in Molecular/Biology/Genetics journals that are cited for research in Molecular/Biology/Immunology journals. The green path means that research published in Molecular/Biology/Genetics and Psychology/Pedagogy/Sociology journals is often cited in Medicine/Medical/Clinical journals.

A total of 111 research areas were identified in this study. The top five research areas with outstanding representation were shown in Table 3. The most representative research field is Integrative and Complementary Medicine (184 records, 50.69% of all articles), followed by Neurosciences and Neurology (108, 29.75%), Neurosciences (82, 22.58%), Research and Experimental Medicine (54, 14.87%), and Medicine, Research and Experimental (42, 11.57%).

**Table 3:** Top five research areas related to acupuncture treatment of cognitive impairment.

**Rank**	**Research areas**	**Records (*n*)**	**% (of 363)**
1	Integrative and Complementary Medicine	184	50.69
2	Neurosciences and Neurology	108	29.75
3	Neurosciences	82	22.58
4	Research and Experimental Medicine	54	14.87
5	Medicine, Research and Experimental	42	11.57

### Literature co-citation analysis

**Figure 6:**
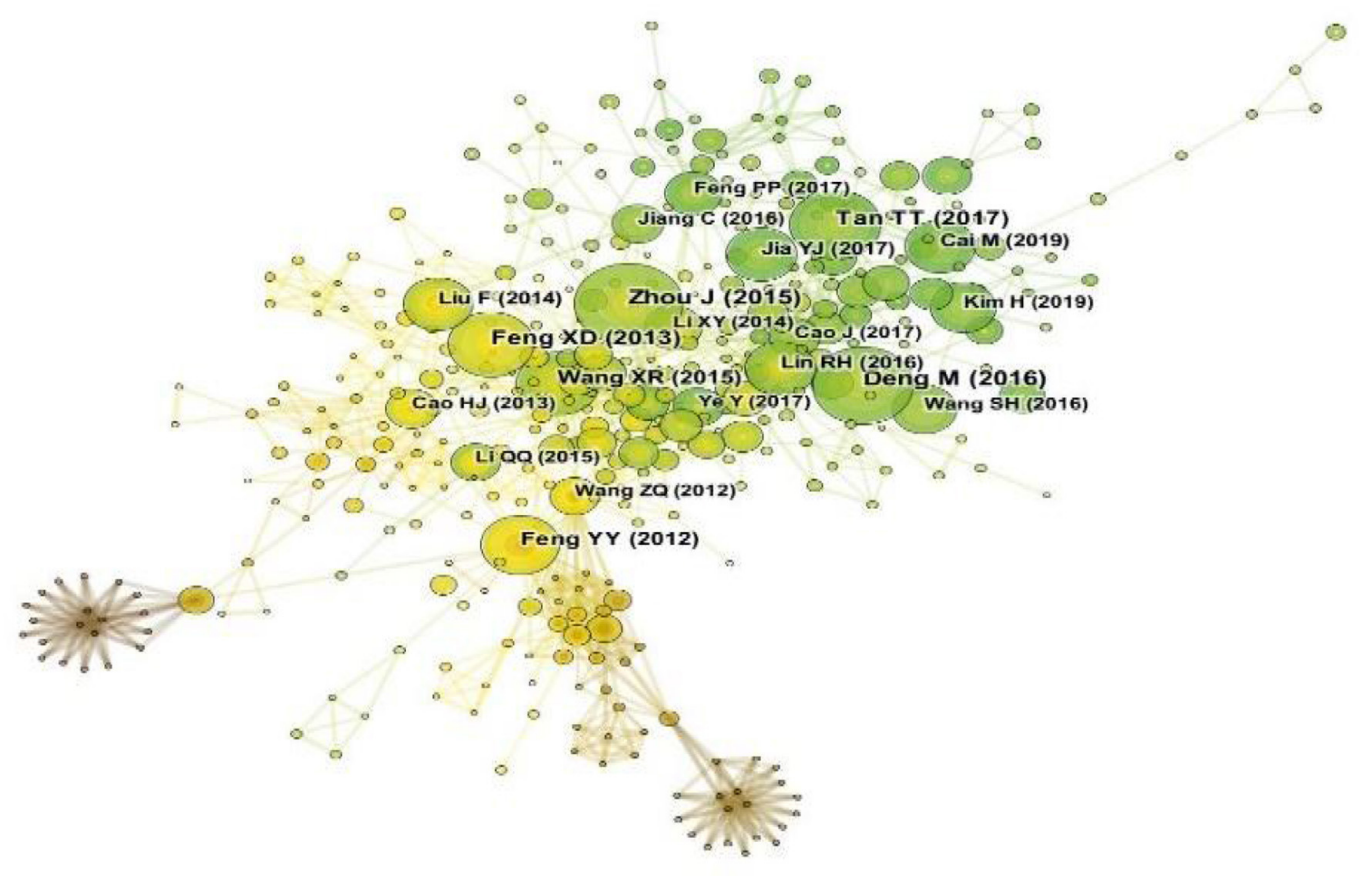
Map of co-citation journals.

The 552 nodes and 1,540 links in the citation map were shown in Figure 6, and the selection criteria were top 20 per slice (Timespan: 2007–2022, slice length = 1 year), were chosen to form the network map of co-cited references using CiteSpace. The top five publications in terms of co-citation frequency and centrality were shown in Table 4. By analyzing co-citation frequency and centrality, this study provided fundamental data on the research on acupuncture for cognitive impairment.

**Table 4:** Top five publications for co-citation and centrality.

**Rank**	**Co-citation counts**	**Co-citation references**	**Rank**	**Centrality**	**Co-citation references**
1	23	Zhou et al., 2015	1	0.24	Feng et al., 2015
2	22	Deng and Wang 2016	2	0.18	Wang et al., 2015
3	20	Tan et al., 2017	3	0.11	Chen et al., 2015
4	19	Feng et al., 2013	4	0.09	Lin et al., 2015
5	18	Wang et al., 2015	5	0.09	Dhond et al., 2008

The first article was published in 2015 by Zhou et al. (2015), which demonstrated that acupuncture can enhance the effect of drugs in improving cognitive function in AD treatment, and improve the ability of daily living in AD patients.

The second article was published in 2016 by Deng and Wang (2016), which used a meta-analysis of randomized controlled trials to estimate the clinical effectiveness and safety of acupuncture for AMCI.

The third article was published in 2017 by Tan et al. (2017), which used acupuncture at Tiaoshen Yizhi acupoints can regulate brain networks by increasing connectivity between cognition-related regions, thereby improving cognitive function in patients with mild cognitive impairment.

The fourth article was published in 2013 by Feng et al. (2013), which suggested that inhibition of NF-κB-mediated neuronal cell apoptosis may be one mechanism *via* which electroacupuncture at Baihui and Shenting exerts a therapeutic effect on post-stroke cognitive impairment.

The last article was published in 2015 by Wang et al. (2015), which provided evidence that the neuroprotection of acupuncture in models of vascular dementia was *via* the Nrf2 activation and Nrf2-dependent microglia activation.

### Author cooperation network analysis

The author cooperation network map is constructed through VOSviewer, which can display the cooperation relationship between authors. The top five most active authors were showed in Table 5. Tao Jing (23 documents) and Liu Cun-Zhi (23 documents) ranked first, followed by Chen Li-Dian (18 documents), Yang Jing-Wen (17 documents), and Huang Jia (15 documents).

**Table 5:** Top five active authors and cited authors.

**Rank**	**Active authors**	**Documents (*n*)**	**Cited authors**	**Citations (*n*)**
1	Tao Jing	23	Tao Jing	403
2	Liu Cun-Zhi	23	Liu Cun-Zhi	399
3	Chen Li-Dian	18	Yang Jing-Wen	300
4	Yang Jing-Wen	17	Chen Li-Dian	287
5	Huang Jia	15	Wang Xue-Rui	284

**Figure 7:**
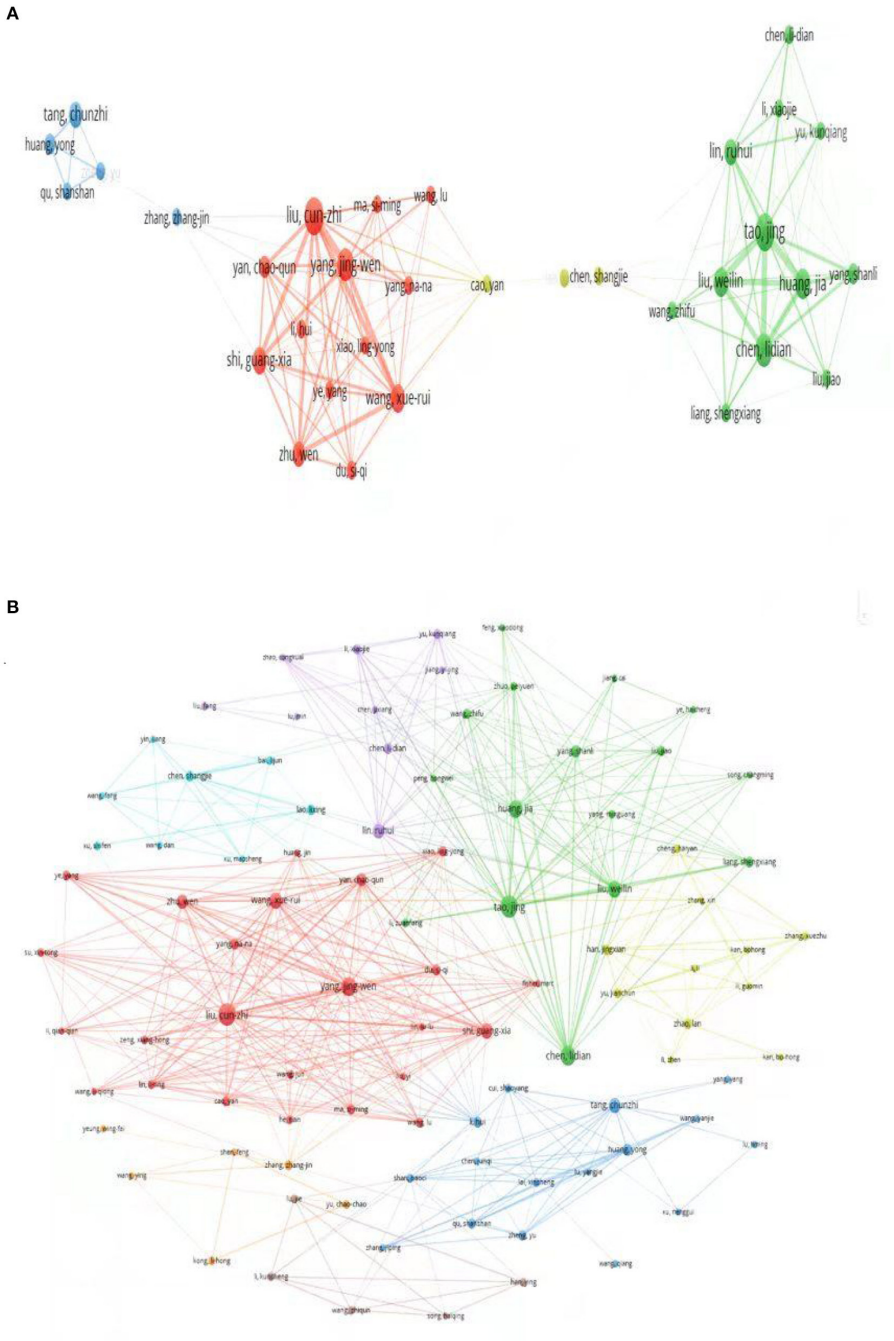
Author cooperation network analysis. **(A)** Co-authorship network map among authors with more than 5 publications. **(B)** Co-citation network map among authors.

The collaborative network between authors who have published more than five papers is shown in Figure 7A. The co-citation network of 125 authors is shown in Figure 7B. Table 5 also shows the top five authors cited. Tao Jing (403 citations) ranked first, followed by Liu Cun-Zhi (399 citations), Yang Jing-Wen (300 citations), Chen Li-Dian (287 citations), and Wang Xue-Rui (284 citations).

### Keyword analysis

**Figure 8:**
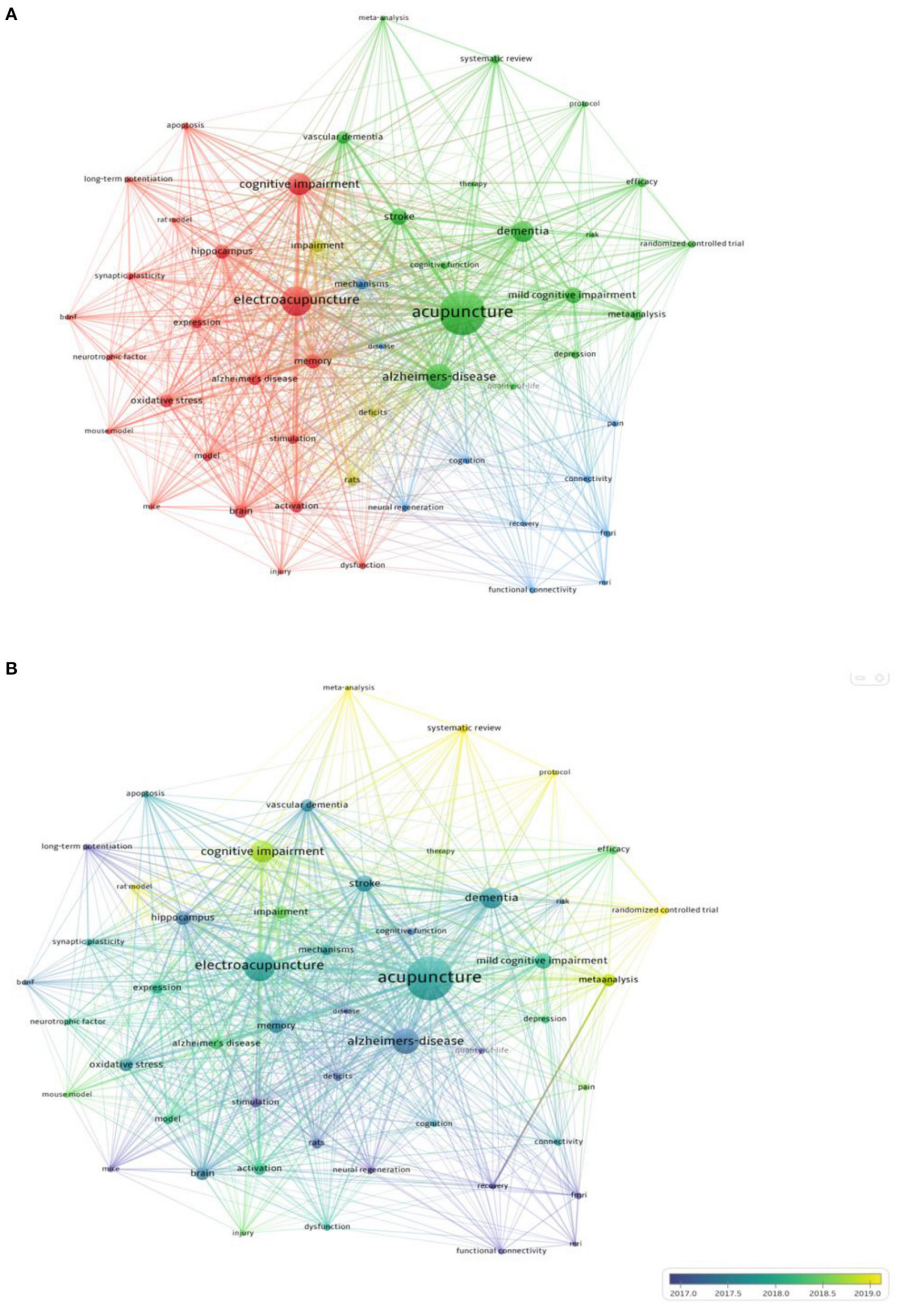
Analysis of keywords. **(A)** Co-occurrence network of keywords. **(B)** Analysis of keywords by average publication year (blue: earlier, yellow: later).

**Figure 9:**
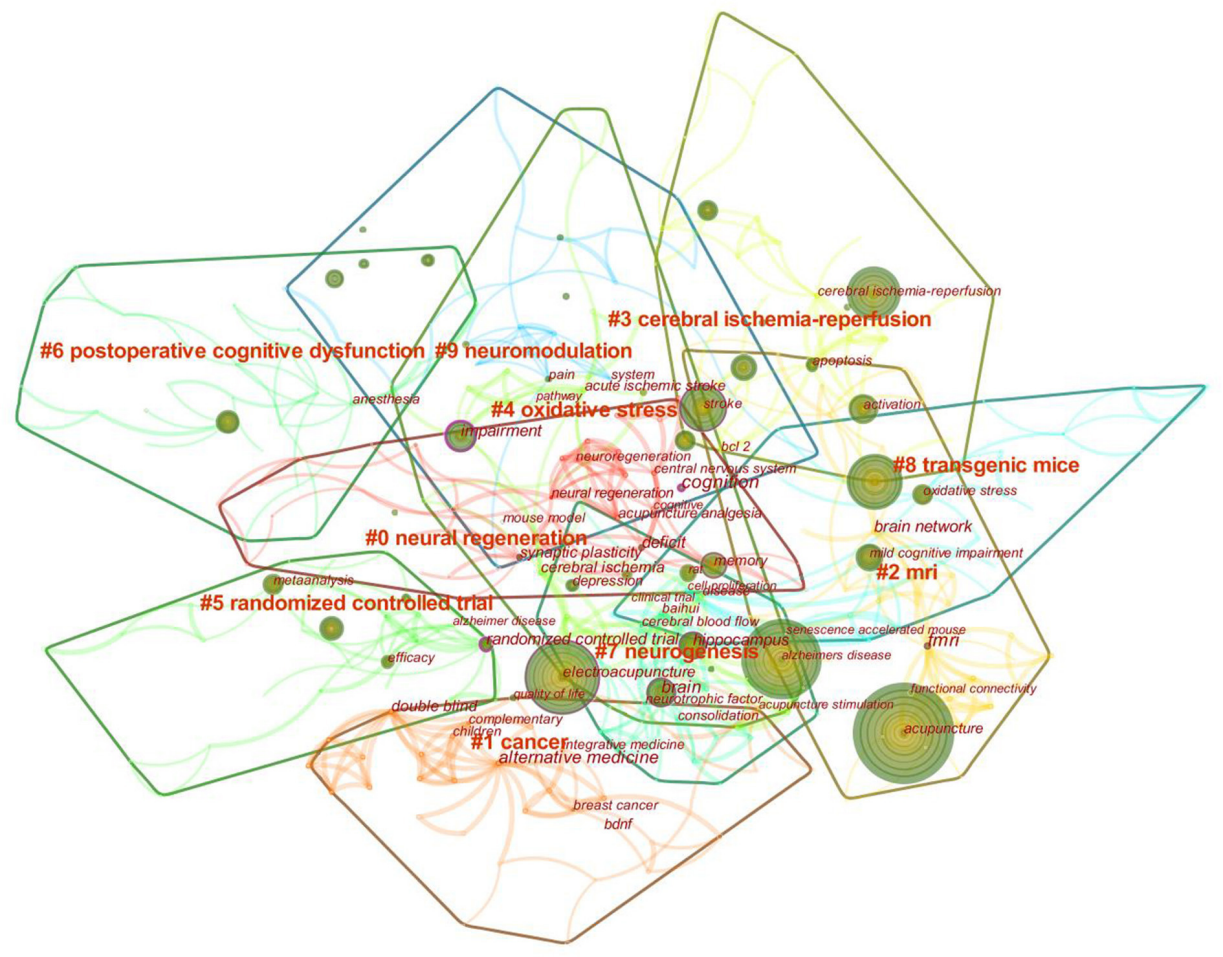
Clustering of keyword.

**Figure 10:**
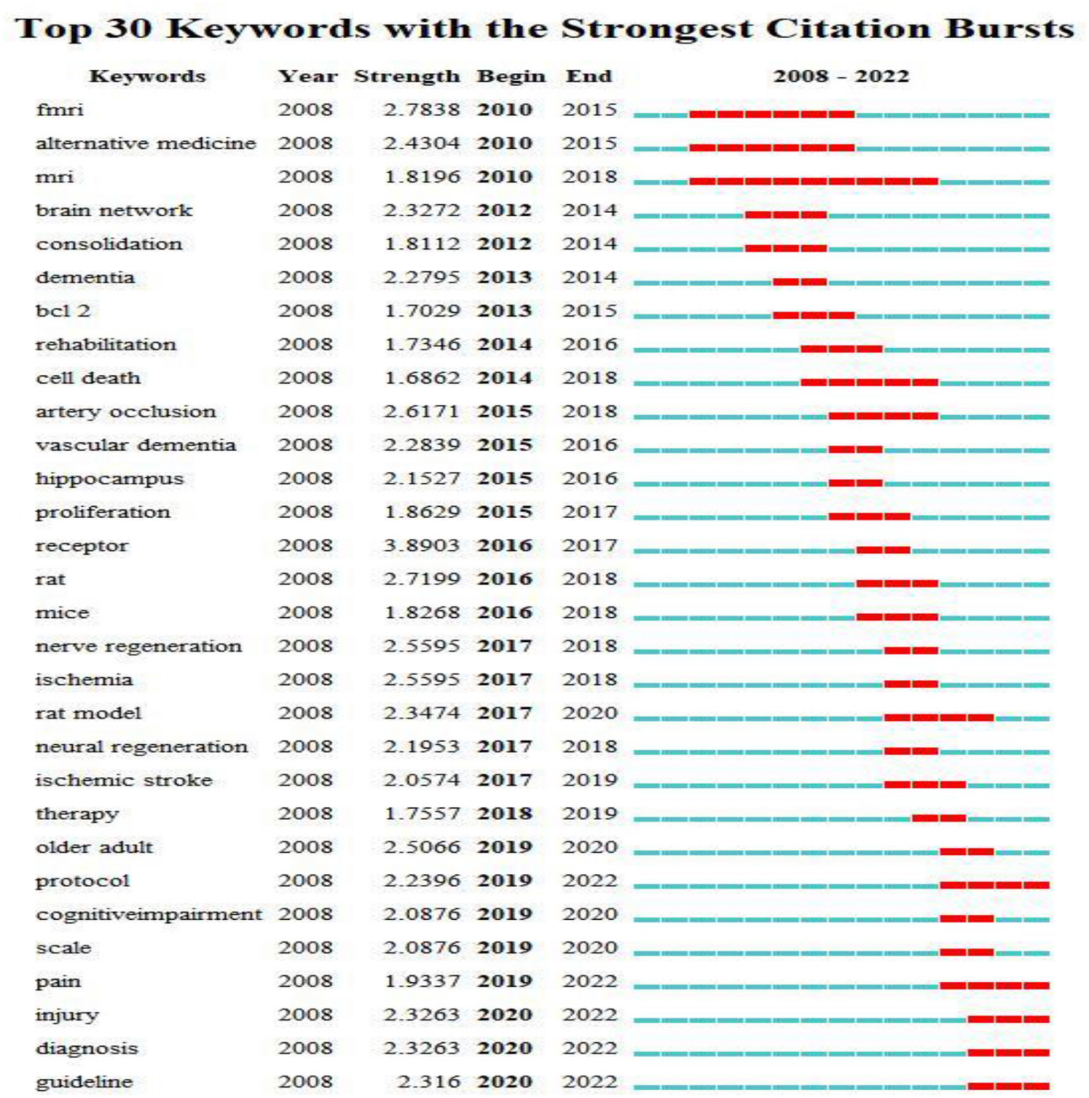
Top 30 keywords with the strongest citation bursts. The red bars mean the keywords occurred frequently; the green bars mean the keywords occurred infrequently. A greater strength indicates a higher frequency of occurrence.

A keyword is a summary of a research topic. Through the analysis of keywords, we can learn about research hotspots in specific fields and predict future development trends. The high-frequency keywords for acupuncture treatment of cognitive impairment were shown in Table 6. Visual analysis of these keywords using VOSviewer found a total of 51 keywords that appeared more than ten times in Figure 8A. The identified keywords were displayed by VOSviewer according to the average publication year, as shown in Figure 8B. On the basis of the co-occurrence of keywords, they are clustered by the algorithm of CiteSpace, and ten keyword cluster labels can be obtained, the results of keyword clustering are shown in Figure 9. Among them, cluster #0 is concentrated in 2017, with a small temporal distance and a high profile. Observing this type of literature, it can be seen that neural regeneration is receiving attention from researchers. CiteSpace is used to detect emergent burst keywords, which are identified as indicators of emerging trends, as shown in Figure 10. After 2018, the most cited keywords in this field were “protocol,” “pain,” “injury,” “diagnosis,” and “guideline”.

**Table 6:** Top five keywords related to acupuncture treatment of cognitive impairment.

**Rank**	**Keyword**	**Records (*n*)**	**Rank**	**Keyword**	**Centrality**
1	Alzheimer's disease	112	1	Brain	0.19
2	Stroke	54	2	Activation	0.16
3	Memory	42	3	Memory	0.12
4	Vascular dementia	34	4	Mechanism	0.09
5	Oxidative stress	31	5	Randomized controlled trial	0.09

## Discussion

### General trends in research fields from 2007 to 2022

Research on the treatment of cognitive impairment with acupuncture has been paid more and more attention. The total number of annual publications has steadily increased over the past 15 years. Furthermore, annual publication production is expected to increase rapidly over the next decade, suggesting a bright future for this field of research. Countries that have made great contributions to the publication of the article are China, the USA, South Korea, Germany and England. The USA, China and England have cooperated with various countries to a certain extent in this field. In terms of the top five productive institutions, a total of 145 articles, among them, Beijing Univ Chinese Med (38 papers), Capital Med Univ (38 papers). In terms of centrality, Chinese Acad Sci (0.27) ranks first, followed by Beijing Huairou Dist Hosp Tradit Chinese Med (0.23), and Univ Hong Kong (0.23). Chinese Acad Sci with the highest centrality (0.27) shows that it works closely with many institutions. Co-author analysis by countries and institutions shows that international cooperation is becoming a trend in this field, but the breadth and intensity of cooperation are not ideal, for example, the USA and China have little cooperation. From the perspective of research institutions, most cooperative institutions are limited to internal connections, and transnational cooperation is relatively rare, this situation has caused certain obstacles to the development of this field. Therefore, it is recommended that the USA, China and other countries actively cooperate and communicate to promote the research and development of acupuncture for cognitive impairment in neuroscience.

Judging from the distribution of journals and co-authored academic journals, the top five journals published 109 articles, accounting for 32.5% of all articles. The *Evid-Based Complem Alternm* has the largest number of published articles (34, or 10.4% of all articles). It shows that this kind of complementary and alternative therapy is gradually being welcomed by people. In addition, journal co-citation analysis reveals influential research in the field with high citation rates, *BMC Complem Altern* has the most citations (395), which is representative. However, research on cognitive impairment is also potentially valuable based on other journals. For example, it also involves the fields of molecular science, biological science, immunology, as well as the fields of mental health, education and society. A dual map overlay representing the distribution of academic journal topics was shown in Figure 5. Considering the three main approaches in the figure, research on acupuncture treatment of cognitive impairment is biased toward basic research and has gradually transformed the main research results into clinical research.

Literature co-citation network research can explore the development and evolution of a discipline. According to the five references cited, it is shown that acupuncture modulates the brain network both by enhancing the connections between cognitively relevant regions; It can also improve cognitive impairment after stroke by inhibiting NF-κB-mediated neuronal apoptosis; In addition, activation of Nrf2 and Nrf2-dependent microglia by acupuncture can exert a neuroprotective effect in a vascular dementia model. It shows that acupuncture can improve cognitive impairment through various regulation methods.

Author collaboration networks and co-citation analysis can reveal influential authors and potential collaborations in the field. Tao Jing, Liu Cun-Zhi, Chen Li-Dian and Yang Jing-Wen are all included in the top five active and cited authors. Tao Jing's team showed through basic experiments that electroacupuncture can improve cognitive impairment caused by Alzheimer's disease (AD) by up-regulating BDNF in APP/PS1 transgenic mice (Lin et al., 2018). In addition, she showed in a recent study that electroacupuncture could enhance oxyglycolysis (AG) by activating AMPK in the early stages of AD, thereby improving cognitive function in AD. To investigate whether dopamine (a key mediator of synaptic plasticity) is involved in the improvement of cognitive impairment, Liu Cun-Zhi et al. used a model of vascular dementia in male Wistar rats with bilateral common carotid artery occlusion, it was found that acupuncture at Zusanli (ST36) and Baihui (GV20) in rats with vascular occlusion can enhance synaptic plasticity in the hippocampus by activating D1/D2 receptors in rats with vascular occlusion, thereby reducing cognitive impairment. Chen Li-Dian and Yang Jing-Wen conducted randomized controlled trials of acupuncture for cognitive impairment, respectively, the results show that acupuncture, as a complementary and alternative therapy, has fewer side effects and various acupuncture methods, etc, and has played a positive role in improving the cognition and daily performance of patients with cognitive impairment.

From the author's cooperation network analysis map, red star clusters indicate Beijing Univ Chinese Med, Acupuncture Res Ctr, Liu Cun-Zhi et al. with Capital Med Univ, Acupuncture and Moxibust Dept, Yang Jing-Wen and others. The green clusters indicate the collaboration between Fujian Univ Tradit Chinese Med, Coll Rehabil Med Tao Jing et al. and Natl Local Joint Engn Res Ctr Rehabil Med Technol, Chen Li-Dian. The blue clusters indicate that Guangzhou Univ Chinese Med, Med Coll Acu Moxi and Rehabil Tang Chun-Zhi collaborated with Huang Yong et al. of Southern Med Univ, Sch Tradit Chinese Med. From the perspective of author collaboration networks, the top five active authors are limited to domestic connections, and there are relatively few cross-border collaborations, which hinders the development of this field. Therefore, relevant authors are advised to actively carry out cross-border cooperation and communication to promote the research and development of cognitive impairment.

The five most frequently used keywords were “Alzheimer's disease,” “stroke,” “memory,” “vascular dementia,” and “oxidative stress.” The five keywords in terms of centrality were “brain,” “activation,” “memory,” “mechanism,” and “randomized controlled trial.” Complementary and alternative medicine has become a promising option for the treatment of cognitive impairment, For example, a systematic review pointed out that acupuncture is more effective than drugs or conventional means in the treatment of mild cognitive impairment and dementia [odds ratio = 1.39; 95% confidence interval (CI): 1.24,1.56] (He et al., 2021). Furthermore, in a meta-analysis, it was shown that non-pharmacological interventions have certain potential for the treatment of AD, such as acupuncture treatment, exercise intervention, music therapy, cognitive intervention, and repetitive meridian magnetic stimulation (rTMS) (Wang et al., 2020). Neurons in the hippocampus play a very important role in learning and memory, the proliferation and differentiation of neurons are accompanied by the process of learning and memory changes (Huang et al., 2015; Mattfeld and Stark, 2015). Studies have shown that the reduction of hippocampal CA1 and CA3 neurons is associated with cognitive deficits in these specific regions (Hammerschmidt et al., 2013; Yeung et al., 2014). Therefore, protecting the structural integrity of CA1 and CA3 regions is beneficial to increase the number of surviving neurons and thus improve learning and memory. In recent years, both basic research and clinical practice have shown the advantages of acupuncture in the treatment of cognitive impairment, for example, in a randomized controlled trial, acupuncture treatment slowed cognitive decline and enhanced hippocampal functional connectivity (FC) (Li et al., 2020), showing long-term effects of acupuncture on cognitive impairment. The five new keywords that have emerged in recent years are: protocol, pain, injury, diagnosis and guidelines, it indicates that research on this topic is gradually moving toward more systematic, standardized trial protocols and guidelines.

From the research trends, it can be seen that the future research on acupuncture treatment of cognitive impairment will continue to grow. This study had a bird's eye view of the acupuncture for cognitive disorders current research frontiers In addition, it is one of the pioneering studies to identify countries, institutions, journals, authors, references that have played important roles in global cognitive impairment research. The conclusions of our research provide suggestions of promising domains that acupuncture can be applied to and point out existing concerns in the recent literature. With detailed exploration of the literature with vivid figures and appropriate tables, we hope this study would be beneficial to all readers of related scholars, doctors and Rehabilitation therapist etc.

### Advantages and limitations

This is a bibliometric analysis to examine trends in acupuncture treatment of cognitive impairment research. This paper uses two well-known scientometric software tools (CiteSpace and VOSviewer) to construct and visualize bibliometric networks by analyzing co-authorship, co-citation, co-occurrence and citation bursts. However, this study has certain limitations. First, this study mainly focuses on quantitative analysis, but less on qualitative analysis. Second, the retrieval is mainly performed in the Web of Science database, it would be better if combined with other databases like PubMed and Scopus. However, it is worth noting that Web of Science is the most commonly used scientometrics database, and visualization-based literature analysis also lays a foundation for researchers to quickly understand the hotspots and trends in this field.

## Conclusion

This bibliometric study can help researchers identify the current status and emerging trends in the field of acupuncture for cognitive impairment over the past 15 years, at the same time, it also provides a perspective on the development trend and hot topic of acupuncture therapy cognitive impairment. CiteSpace and VosViewer are just a software that can be visual and network analysis, we have roughly analyzed research hotspots. In the future, we need more rigorous clinical trials and more research on related mechanisms.

## Data availability statement

The raw data supporting the conclusions of this article will be made available by the authors, without undue reservation.

## Author contributions

C-CN and K-QS conceived the idea. JY, X-DR, and Y-FF completed the data collection. ZL, X-LS, M-YY, and S-KQ conducted the data analysis. C-CN drafted the manuscript. X-DF, JG, and X-LS revised the manuscript. All authors were involved in the interpretation of the study findings and contributed to the development of the protocol. All authors have critically reviewed, provided intellectual input to the manuscript, and approved the final version of the manuscript.

## Funding

This research was supported by the National Natural Science Foundation of China (Grant Number 82174473), National Natural Science United Fund Project (Grant Number U2004131), and Henan Provincial Nurnent Repair Key Laboratory Open Project (Grant Number HNSJXF-201-005).

## Conflict of interest

The authors declare that the research was conducted in the absence of any commercial or financial relationships that could be construed as a potential conflict of interest.

## Publisher's note

All claims expressed in this article are solely those of the authors and do not necessarily represent those of their affiliated organizations, or those of the publisher, the editors and the reviewers. Any product that may be evaluated in this article, or claim that may be made by its manufacturer, is not guaranteed or endorsed by the publisher.
